# Clinical management and mortality among COVID-19 cases in sub-Saharan Africa: A retrospective study from Burkina Faso and simulated case analysis

**DOI:** 10.1016/j.ijid.2020.09.1432

**Published:** 2020-12

**Authors:** Laura Skrip, Karim Derra, Mikaila Kaboré, Navideh Noori, Adama Gansané, Innocent Valéa, Halidou Tinto, Bicaba W. Brice, Mollie Van Gordon, Brittany Hagedorn, Hervé Hien, Benjamin M. Althouse, Edward A. Wenger, André Lin Ouédraogo

**Affiliations:** a Institute for Disease Modeling, Bellevue, WA, USA; b IRSS-Clinical Research Unit of Nanoro, Burkina Faso; c Ministry of Health, Teaching Hospital Yalgado Ouedraogo, Ouagadougou, Burkina Faso; d Centre National de Recherche et de Formation Sur le Paludisme, National Public Health Institute, Ouagadougou, Burkina Faso; e Centre des Operations de Réponses aux Urgences Sanitaires, Ouagadougou, National Public Health Institute, Burkina Faso; f Centre MURAZ, National Public Health Institute, Ouagadougou, Burkina Faso; g IRSS, Programme de Recherche Sur les Politiques et les Systèmes de Santé, Bobo-Dioulasso, Burkina Faso; h University of Washington, Seattle, WA, USA; i New Mexico State University, Las Cruces, NM, USA

**Keywords:** Sub-Saharan Africa, Burkina Faso, Mortality, SARS-CoV-2 infection, Clinical management of SARS-CoV-2 infection: convalescent plasma, Oxygen therapy, Health systems strengthening

## Abstract

**Background:**

Absolute numbers of COVID-19 cases and deaths reported to date in the sub-Saharan Africa (SSA) region have been significantly lower than those across the Americas, Asia and Europe. As a result, there has been limited information about the demographic and clinical characteristics of deceased cases in the region, as well as the impacts of different case management strategies.

**Methods:**

Data from deceased cases reported across SSA through 10 May 2020 and from hospitalized cases in Burkina Faso through 15 April 2020 were analyzed. Demographic, epidemiological and clinical information on deceased cases in SSA was derived through a line-list of publicly available information and, for cases in Burkina Faso, from aggregate records at the Centre Hospitalier Universitaire de Tengandogo in Ouagadougou. A synthetic case population was probabilistically derived using distributions of age, sex and underlying conditions from populations of West African countries to assess individual risk factors and treatment effect sizes. Logistic regression analysis was conducted to evaluate the adjusted odds of survival for patients receiving oxygen therapy or convalescent plasma, based on therapeutic effectiveness observed for other respiratory illnesses.

**Results:**

Across SSA, deceased cases for which demographic data were available were predominantly male (63/103, 61.2%) and aged >50 years (59/75, 78.7%). In Burkina Faso, specifically, the majority of deceased cases either did not seek care at all or were hospitalized for a single day (59.4%, 19/32). Hypertension and diabetes were often reported as underlying conditions. After adjustment for sex, age and underlying conditions in the synthetic case population, the odds of mortality for cases not receiving oxygen therapy were significantly higher than for those receiving oxygen, such as due to disruptions to standard care (OR 2.07; 95% CI 1.56–2.75). Cases receiving convalescent plasma had 50% reduced odds of mortality than those who did not (95% CI 0.24–0.93).

**Conclusions:**

Investment in sustainable production and maintenance of supplies for oxygen therapy, along with messaging around early and appropriate use for healthcare providers, caregivers and patients could reduce COVID-19 deaths in SSA. Further investigation into convalescent plasma is warranted until data on its effectiveness specifically in treating COVID-19 becomes available. The success of supportive or curative clinical interventions will depend on earlier treatment seeking, such that community engagement and risk communication will be critical components of the response.

## Highlights

• Countries within the sub-Saharan Africa (SSA) region may experience high COVID-19 case fatality rates.
• Over the first months of the epidemic in SSA, deceased cases have tended to mainly be male, aged >50 years and have underlying comorbidities.
• Delayed or no care-seeking was prevalent among deceased COVID-19 cases in Burkina Faso.
• Analysis on a synthetic case population suggested that treatment with oxygen therapy or convalescent plasma reduced the adjusted odds of COVID-19 mortality.
• Low-cost, scalable and sustainable strategies for COVID-19 case management in the SSA context warrant attention and investment to reduce disparity in case fatality.

## Introduction

At 6 months after SARS-CoV-2 (COVID-19) was introduced into the region, countries across sub-Saharan Africa (SSA) were reporting significantly fewer cases than higher income countries and some low-middle-income countries (LMICs) on other continents (World Health Organization, 2020b). As a result, the majority of evidence to date reflects disease progression, hospitalization rates and deaths among cases in more developed and higher transmission settings across Europe, North America (CDC COVID-19 Response Team, 2020) and Asia (Chen et al., 2020, Guan et al., 2020). While absolute case and death counts are lower in SSA, individual countries are reporting high case fatality ratios (CFRs) and the available information warrants investigation into mortality reduction strategies. Given the limited healthcare capacity to manage critical cases and poor detection leading to delayed care-seeking (Africa Center for Strategic Studies, 2020, Gilbert et al., 2020, Skrip et al., 2020) it has been hypothesized that the SSA region is vulnerable to high rates of COVID-19 deaths.

Healthcare-related resource constraints across SSA range from inadequate supplies of medical equipment to low per capita capacity for isolation and treatment. A recent report estimated that the entire African continent has 1% of the ventilator capacity of the United States (Maclean and Marks, 2020). Furthermore, countries have reported between one and 36 hospital beds per 10,000 capita (World Health Organization, 2020d). These constraints have been at odds with policies to quarantine presumptive cases in precautionary isolation centers (allAfrica, 2020a, allAfrica, 2020b) or isolate all positive cases in dedicated hospitals as a control strategy (World Health Organization, 2020c). Moreover, messaging around the lack of effective treatment for COVID-19 or unavailability of resources may be deterring early care-seeking among individuals who develop symptoms and could benefit from supportive care. Given the systemic vulnerabilities in health system capacity, it is critical to investigate and invest in lower cost and readily scalable interventions, including some already with precedent for reducing the morbidity and mortality associated with respiratory and/or viral infections. Notably, oxygen therapy (Geiling et al., 2014) and use of blood-related therapies (Office of the Commissioner, 2020) hold potential for not only managing COVID-19 cases but also treating other conditions prevalent or emerging in LMICs.

**Figure 1:**
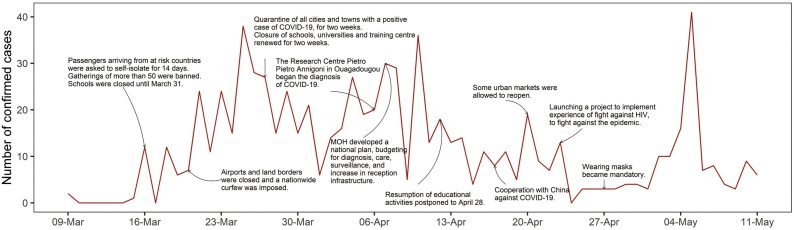
COVID-19 epidemic curve (weekly cases in red) and timeline of control efforts in Burkina Faso 09 March 2020–11 May 2020. The first case in Burkina Faso was detected on 09 March 2020. The Centre for Research Centre Pietro Annigoni (CERBA) in Ouagadougou initiated diagnosis of COVID-19 on 06 April, and as of 21 April the Centre has analyzed 460 samples. Two more laboratories in Ouagadougou–The Yalgado Hospital Centre and the National Public Health Laboratory–also analyzed the same samples to ensure quality of results. On 07 April, the Minister of Health in Burkina Faso presented a national plan to respond to the COVID-19 pandemic. In this plan, budgets were specifically allocated to diagnosis, surveillance, infection prevention and control, patient safety, research, and developing remote quick intervention equipment.

Burkina Faso was among the first countries in SSA to report COVID-19 cases. The first case of COVID-19 in Burkina Faso was confirmed on 09 March 2020. Since then, several containment measures have been adopted by the government of Burkina Faso, including social distancing, closure of airports and land borders, quarantine of the affected cities and the mandatory use of masks (Figure 1). Such efforts may have suppressing transmission, yet the country is reporting among the highest numbers of deaths regionally.

COVID-19 cases in Africa currently comprise approximately 1% of the reported global burden (World Health Organization, 2020a). Low levels of detection, partially due to insufficient testing capacity and laboratory supplies, and the lack of published data from individual African countries challenge understanding of the true scale of infection as well as study of potential risk factors and intervention approaches (Skrip et al., 2020). The current study used existing, yet minimal, data to generate insight into the impact of potentially lower cost and more logistically feasible approaches to case management in the LMIC context, where care-seeking may be affected by the perceived lack of treatment options. An overview of demographic and clinical characteristics of deceased cases across SSA overall and in Burkina Faso specifically was provided. Trends in mode of detection and care-seeking among all cases were compared with trends among cases who ultimately died of the disease. Mitigation strategies to prevent excess mortality, while also preparing for future treatment opportunities such as the use of blood-related products, were investigated through statistical analysis of a synthetic case population matched to demographic and clinical characteristics of cases in West Africa.

## Methods

### Data

Data from publicly available sources were line-listed to assess epidemiological and clinical characteristics of deceased COVID-19 cases across the entire SSA region. Specifically, data on sex, age, underlying conditions, and mode of detection were identified for deceased cases, as previously performed for a line list being curated for all cases in SSA (Skrip et al., 2020). Additionally, data on age, sex and time in hospital were recorded for 49 deceased cases reported as of 10 May 2020 and 163 hospitalized cases reported as of 15 April 2020, from the Centre Hospitalier Universitaire (CHU) de Tengandogo in Ouagadougou, Burkina Faso. Data were presented as frequencies and percentages. Age distributions between hospitalized and deceased cases were compared using a Chi–squared test.

### Data simulation and statistical analysis

A synthetic case study population of individual COVID-19 cases was generated using multinomial distribution sampling (Ikeda et al., 2011, Bai et al., 2013, Xu et al., 2017). Specifically, for a fixed stratum n and a vector of proportions π = (π1,π2,…,πk), X ∼ Mult (n, π) was sampled to attain a sample representative of characteristics (age, gender, comorbidity, disease outcome) in the population. The base uninfected population was randomly drawn using demographics as reported by census data to account for age and gender strata in Burkina Faso (Institute for Health Metrics and Evaluation, 2015). Age-adjusted distributions of non-communicable diseases (i.e., hypertension and diabetes) in West African populations were used to randomly assign underlying conditions to individuals (Agaba et al., 2017, Soubeiga et al., 2017). In the absence of age data for all confirmed cases in Burkina Faso, COVID-19 infection was randomly assigned to individuals following the age-binned distribution of cases in Senegal (Ministère de la Santé et de l’Action sociale, 2020), assuming that the infection dynamics in the two countries were similar. Age distributions of COVID-19 hospitalization and mortality rates were based on data from the CHU in Burkina Faso. Hospitalization and mortality rates in younger and elderly ages were assumed to vary 100% and 50%, respectively, to address uncertainty in measurement and propagate it using non-linear combinations estimates of errors.

To evaluate COVID-19 case fatality rates in the absence of oxygen therapy, this study used adjusted odds ratios as increased risk of death by hypoxia (hypoxia is defined with a cut-off for oxygen saturation rate SpO_2_ <90%) in patients with acute lower respiratory infections (Lazzerini et al., 2015) and brain injury (Seo et al., 2019) to simulate a binomial distribution of oxygen treatment and non-treatment, as described in the contingency Table (Supplementary Table 1).

To consider a reduction in COVID-19 case fatality rates in the presence of convalescent plasma therapy, a convalescent plasma treatment study was simulated in the hypothetical inpatient population. Given the current lack of data on anti-COVID-19 convalescent plasma treatment, it was assumed that there was an absolute risk difference in mortality of 10.5% between treatment and non-treatment groups, considering that convalescent plasma during the current COVID-19 pandemic is two-fold less likely to reduce mortality than convalescent plasma during the 1918 Spanish flu pandemic (21%; 95% CI 15–27%) (Luke et al., 2006). Distribution of sample sizes to meet a 10.5% risk difference in between-groups mortality is described in Supplementary Table 1; hospitalized patients were randomly assigned to the groups.

Associations between the odds of survival and patient characteristics (age, gender and comorbidity), convalescent plasma treatment and healthcare capacity (oxygen therapy) on COVID-19 mortality were estimated using logistic regression. The adjusted odds ratio for survival among cases receiving intervention (oxygen therapy or convalescent plasma) versus those not receiving the intervention were calculated with 95% confidence intervals.

### Ethics statement

All data presented here were derived from publicly available sources or provided by CHU as part of the national epidemic response program. The data from CHU were reported in aggregate, as frequency distributions across 10-year age bins, by sex or length of hospital stay. The study was therefore not subject to review by an ethics board.

## Results

### Data description

**Figure 2:**
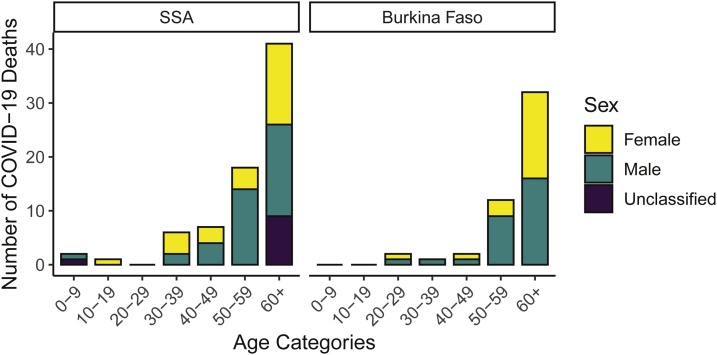
Distribution of sex and age among reported deceased COVID-19 cases in sub-Saharan Africa (SSA) overall (excluding Burkina Faso) and in Burkina Faso.

As of 10 May 2020, 976 deaths had been reported out of the 39,104 confirmed COVID-19 cases across 48 countries in the SSA region (regional CFR 2.5%) (World Health Organization, 2020a). Line-listed, country-reported data on age and/or sex were available for 75 and 103 deceased cases, respectively. Deceased cases were mainly male (63/103, 61.2%) and had a median age of 62 years (range 28 days–89 years) (Figure 2). Over 78% of deaths were reported among individuals aged ≥50 years (59/75, 78.7%). For those cases with available information, the majority of deaths was among individuals who self-presented to facilities or were tested post-mortem (20/30, 66.7%), rather than among cases that were being followed as part of active monitoring initiatives.

In Burkina Faso, specifically, 49 deaths occurred among 751 detected cases as of 10 May 2020. With the exception of sex, which was reported for all 49 deceased cases, data for analysis were available for 163 hospitalized cases and 33 deaths through 15 April 2020. The majority of deceased cases were male (28/49, 57.1%) and nearly all deaths occurred among individuals aged ≥50 years (30/33, 90.9%) (Figure 2). Underlying conditions were prevalent among deceased cases. Nearly half had a history of hypertension (15/33, 45.5%), diabetes (7/33, 21.2%) or other cardiovascular or pulmonary conditions, such as stroke, embolism or cardiopathy (3/33, 9.1%).

**Figure 3:**
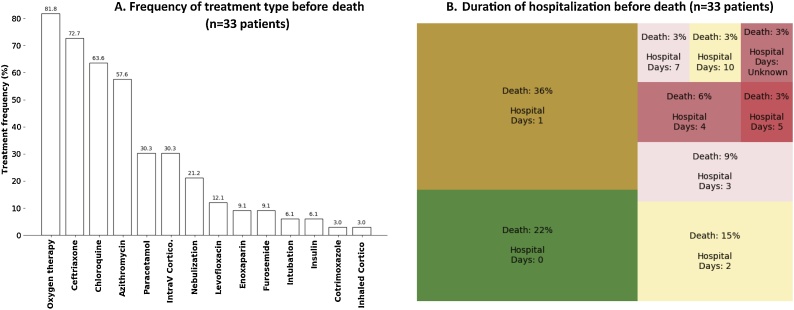
(A) Frequency of treatment type. (B) Length of hospital stay for 33 COVID-19 cases seeking care at CHU de Tengandogo before death in Burkina Faso.

Oxygen therapy was most frequently used to treat hospitalized COVID-19 cases before their deaths (27/33, 81.8%) (Figure 3A). Pulsometer values indicated that 90% of clinical cases presented with a median oxygen level of 61% (IQR 45–82) at time of death, which is below a normal value of 90%. Clinical treatment also routinely included administration of antimicrobials (ceftriaxone, chloroquine and azithromycin) and fever-reducers (paracetamol). Two (2/33, 6.1%) critical patients were intubated before their deaths.

Prior to death, the majority of individuals either did not seek care or were hospitalized for a short duration: 21.9% (7/32) of cases were hospitalized for <1 day or not at all, and 37.5% (12/32) and 15.6% (5/32) were hospitalized for 1 and 2 days, respectively, before death (Figure 3B). The duration of hospitalization for one death was unknown. The age distribution of deceased cases differed from that of all hospitalized cases, with the proportion of hospitalized confirmed cases aged <50 years (56/163, 34.4%) being significantly higher than the proportion of deceased cases <50 years (5/49, 10.2%) (*p* = 0.002).

### Synthetic population simulation and analysis

**Figure 4:**
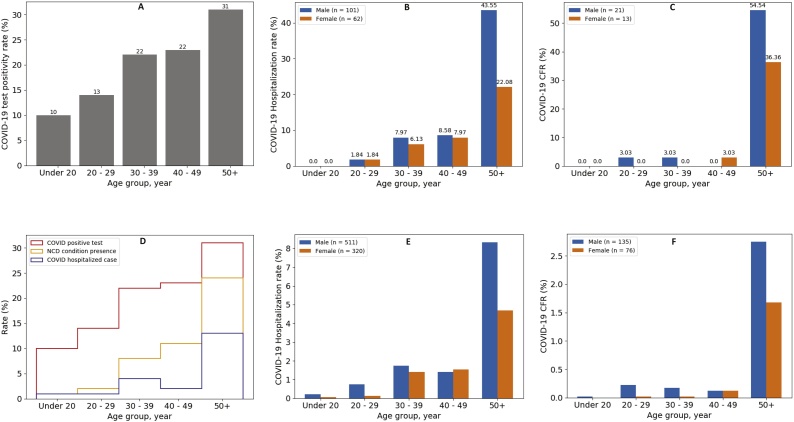
Empirical (Panels A–C) versus simulated (Panels D–F) distribution for age-specific percentages of hospitalizations and underlying conditions in a sample of COVID-19 cases. 10% of the total population was assumed to undergo testing, and stratified random sampling was applied to meet test positivity rates, as observed in Senegal. Hypothetical population sizes by age groups (under 20, 20–29, 30–39, 40–49 and 50+ years) were estimated for SARS-CoV-2 infections (422, 580, 899, 948, 1257, respectively), hospitalizations (11, 36, 129, 121, 534) and deaths (1, 10, 8, 10, 182).

**Figure 5:**
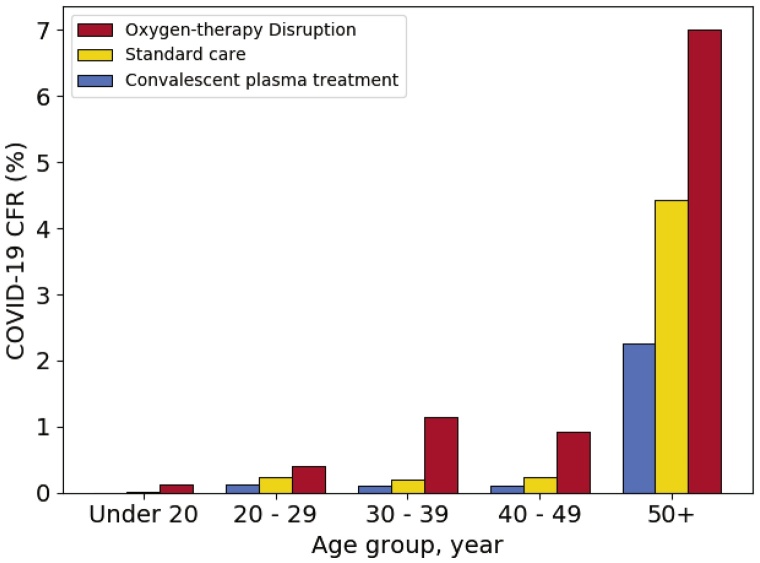
Differential mortality rates modeled as results of convalescent plasma and oxygen therapy effect sizes.

Characteristics of the synthetic case population and distribution of COVID-19 cases are shown in Figure 4. In line with the data from Burkina Faso (Figure 4A–C) and other countries in the region, increased rates of underlying conditions, COVID-19 positivity, inpatient treatment-seeking and death were associated with increasing age. Disruption of oxygen therapy was found to approximately double the case fatality rates across age groups. The introduction of convalescent plasma therapy was associated with reduced COVID-19 case fatality rates, irrespective of age (Figure 5). In the current case population of 831 individuals, the mortality risk difference for those on treatment with convalescent plasma versus not on treatment was 10.5% (95% CI −4 to 25%).

Regression results on the impact of individual factors (age, sex and comorbidities), oxygen disruption and treatment (convalescent plasma) on case fatality rates are shown in Table 1. Age >50 years (AOR = 5.15; 95% CI 3.34–8.17; *p* < 0.001) was associated with significantly increased odds of mortality in the adjusted analysis. No significant association was found between presence of comorbidity (AOR = 1.08; 95% CI 0.77–1.53) or being female (AOR = 0.98; 95% CI 0.69–1.37) in the adjusted analysis. The odds of mortality among COVID-19 cases not treated with oxygen therapy was 2.07 times the odds among cases receiving oxygen therapy (95% CI 1.56–2.75; *p* < 0.001) after adjusting for age, sex and presence of underlying comorbid conditions. Convalescent plasma was found to be significantly (OR 0.5; 95% CI 0.24–0.93; *p* = 0.038) associated with reduction in mortality after adjustment for age, sex and comorbidities.

**Table 1:** Results of logistic regression analysis to assess potential for reduced odds of mortality among cases receiving clinical intervention with oxygen therapy or convalescent plasma.

	Factor		Unadjusted Odd ratio (95%CI)	P-value	Adjusted Odd ratio (95%CI)	P-value
Model I	Age group	Under 50	1.0 (Ref.)		1.0 (Ref.)	
		50+	16.46 (11.24−24.99)	<0.001	16.09 (10.71−24.9)	<0.001
	Gender	Male	1.0 (Ref.)		1.0 (Ref.)	
		Female	0.57 (0.42−0.76)	0.0001	0.54 (0.40−0.73)	<0.001
	Comorbidity	No	1.0 (Ref.)		1.0 (Ref.)	
		Yes	2.44 (1.83−3.28)	<0.001	0.83 (0.58−1.19)	0.310
Model II^a^	Convalescent plasma	No	1.0 (Ref.)		1.0 (Ref.)	
		Yes	0.52 (0.26−0.95)	0.046	0.50 (0.24−0.93)	0.038
Model III^b^	Oxygen	Yes	1.0 (Ref.)		1.0 (Ref.)	
		No	2.04 (1.55−2.7)	<0.001	2.07 (1.56−2.75)	<0.001

a Convalescent plasma therapy’s impact adjusted for age, gender and comorbidity in the context of natural conditions (without oxygen therapy).

b Oxygen therapy’s impact adjusted for age, gender and comorbidity in the context of natural conditions (without convalescent plasma therapy).

## Discussion

This study presents data from a retrospective analysis of clinical and demographic characteristics of deceased COVID-19 cases in SSA. Deaths in the region were more frequent among individuals who self-reported or were tested post-mortem compared with cases identified through active monitoring. This ratio is notably different from that previously identified (Skrip et al., 2020) for the ratio of overall case detection, which was heavily skewed towards detection via active monitoring among all reported cases.

Deceased cases in Burkina Faso tended to be older with a higher proportion of males, relative to all COVID-19 cases hospitalized for treatment. The data further suggest late or no care-seeking among the most severe cases that result in death. This is consistent with observations in other SSA countries. In Liberia, for instance, 11 of 18 reported deaths as of 10 May 2020 occurred in communities and one occurred in a designated COVID-19 facility (National Public Health Institute of Liberia-NPHIL, 2020). Nigeria has also reported clusters of deaths among individuals who were not suspected as having the disease (Daily Trust, 2020a, Daily Trust, 2020b), suggesting that health seeking and access to care are relevant for reducing COVID-19 mortality.

Importantly, the data from Burkina Faso indicate that early detection, oxygen therapy and effective treatments are important for mitigating disease progression and preventing mortality. Using a synthetic case study population matched to data from West Africa, the current study demonstrated the potential effects of appropriate oxygen therapy and convalescent plasma in reducing odds of death among COVID-19 cases. Preliminary evidence has suggested that blood-related products, such as convalescent plasma, may attenuate COVID-19 disease severity or duration (Duan et al., 2020). Convalescent plasma was widely used in previous viral outbreaks (Cheng et al., 2005, Luke et al., 2006, Brown et al., 2017) and the mechanism underlying its effectiveness is well described (Shakir et al., 2010, Rojas et al., 2020). For instance, during the 1918 Spanish Flu pandemic, patients who received convalescent plasma therapy saw a reduced mortality rate, despite treatments being given to more severely ill patients (Luke et al., 2006). Similar results have been estimated for H1N1 flu treatment in smaller studies (Hung et al., 2011). Furthermore, the use of convalescent plasma as treatment in LMIC settings has precedent from the Ebola outbreak in West Africa, where mobile laboratories were used for the apheresis process (Brown et al., 2017).

Of note, the current investigation on convalescent plasma was implemented to reflect the range of uncertainty of its effect size (mortality risk difference of 10.5%, 95% CI −4 to 25%) as observed in studies addressing convalescent plasma’s effectiveness against the Spanish flu. It is important to emphasize that the decision to implement a 10.5% relative risk reduction in mortality with a wide confidence interval was guided by uncertainty around the effect of convalescent plasma in the treatment of COVID-19. Robust data from randomized control trials will allow for better quantification of convalescent plasma effectiveness (i.e. a significant reduction in mortality with less uncertainty) and for informing treatment policies by providing clearer evidence of any risks versus benefits. In parallel with studies investigating clinical effectiveness, proactive efforts are needed for minimizing logistical barriers to implementation (Roback and Guarner, 2020) if recommendations around the use of convalescent plasma are to be adopted. Such efforts might include enhancing blood bank capacity around freezing, storage and donor recruitment to increase supply, and equipping additional sites with transfusion needs to broaden the reach of the intervention and safely monitor (Leider et al., 2010, Roback and Guarner, 2020). Lessons learned on practical challenges can be drawn from LMIC-based clinical trials during the Ebola outbreak (Brown et al., 2017).

The current results are based on parameters informed by the use of oxygen and convalescent plasma on other respiratory diseases (2018 Spanish Flu) and hypoxia (respiratory and brain injury) disease, respectively, such that the findings should be interpreted with caution and revisited as empirical data from the clinical treatments provided to COVID-19 patients become increasingly available. Despite the evidence limitations and practical considerations around implementation, highlighting and evaluating low-cost, potentially scalable approaches for clinical management are critical to lower the CFR and encourage timely care-seeking. This is particularly important in settings with significant undetected community transmission, so that individuals who develop COVID-like symptoms seek care early and are not deterred by the notion that the disease has no cure.

When considering clinical interventions it is important to recognize that availability alone is inadequate to ensure proper clinical care. Reports on oxygen availability in LMICs have indicated that use is common but heterogeneous (Zhu, 2020), with greater availability in more urban environments and at higher levels of care, due to reduced access to pulse oximetry in more rural settings and lower levels of care (PATH, 2018a, PATH, 2018b, PATH, 2018c). In addition to requiring the necessary equipment, patients and caregivers must be willing to accept and administer the care, respectively, which has been observed as a barrier (Dauncey et al., 2019) such as due to perceived cost and lack of familiarity with the equipment, which will need to be addressed during the COVID care response. This is concerning because if patients are escalated to higher levels, this introduces delays in care and means that they will bring COVID with them to hospital populations. In parallel to building treatment capacity, risk communication and community engagement (Bedson et al., 2019) will be essential to increasing awareness of its availability and affordability, so that people will actually seek care promptly. Critical cases who delay care-seeking would likely arrive too late for oxygen or other interventions to have much effect.

If oxygen therapy can reduce the rate of severe disease, and convalescent plasma can reduce progression and mortality, it may be possible to substantially reduce the impact of COVID-19 (Xie et al., 2020) without the need for critical care and respirators, which are more difficult to rapidly scale-up capacity for than these treatments would likely be. Expanding access to oxygen treatment not only benefits COVID patients, but would likely also have long-term impact on child mortality due to pneumonia or all causes (Hansmann et al., 2017); literature has shown risk reductions of about 20–60% in small-scale studies in LMIC (Graham et al., 2017). Investment in capacity and protocols for use could have significant and long-term implications on overall disease burden.

This is the first study of data from deceased COVID-19 cases in the sub-Saharan Africa region. While absolute counts in SSA are considerably lower than in other regions globally, low-cost and readily scalable case management approaches remain important to reducing mortality due to COVID-19 in resource-constrained settings. Investing in capacity for oxygen therapy administration and apheresis, along with research infrastructure to assess effectiveness, could not only improve outcomes during the pandemic response but also have long-term implications for health systems.

## Authors’ contributions

LAS, BMA, EAW and ALO designed the study. LAS, NN, MVG, BH, KD, MK, AG, IV, HT, BWB, and HH collected data for the line list or at the Center Hospitalier Universitaire de Tengandogo in Ouagadougou. ALO conducted the data simulation. LAS and ALO conducted the data analysis. LAS and ALO wrote the first draft of the manuscript. All authors contributed intellectually and made contributions to the manuscript text.

## Conflict of interest

The authors declare no competing interests.
