# Supplementary material for: Clinical management and mortality among COVID-19 cases in sub-Saharan Africa: A retrospective study from Burkina Faso and simulated case analysis
Source: Int J Infect Dis. 2020 Dec;101:194–200. doi: 10.1016/j.ijid.2020.09.1432 (PMC7518969; doi:10.1016/j.ijid.2020.09.1432)
Supplement: Supplementary file 1 [file mmc1.docx]

Supplementary Information

Clinical management and mortality among COVID-19 cases in sub-Saharan Africa: A retrospective study from Burkina Faso and simulated case analysis

Laura Skrip, Karim Derra, Mikaila Kaboré, Navideh Noori, Adama Gansané, Innocent Valéa, Halidou Tinto, Bicaba W. Brice, Mollie Van Gordon, Brittany Hagedorn, Hervé Hien, Benjamin M. Althouse, Edward A. Wenger and André Lin Ouédraogo

**Supplementary Table 1.** Distributions of treatment status and clinical outcome among hospitalized COVID-19 patients in synthetic case population

| Convalescent Plasma |  | Deceased | Not Deceased | Total |
| --- | --- | --- | --- | --- |
|  | Treatment | 12 | 64 | 76 |
|  | Control | 199 | 556 | 755 |
|  | Total | 211 | 620 | 831 |
| Oxygen | Disruption | 235 | 182 | 417 |
|  | Control | 160 | 254 | 414 |
|  | Total | 395 | 229 | 831 |
